# Benchmarking quantum chemical methods with X-ray structures via structure-specific restraints

**DOI:** 10.1107/S2052252525004543

**Published:** 2025-06-17

**Authors:** Birger Dittrich, Rok Breznikar, Gianluca Santarossa, Pamela Whitfield, Henrik Moebitz

**Affiliations:** a Novartis Campus, Novartis Pharma AG, Postfach, BaselCH-4002, Switzerland; b Mathematisch Naturwiss. Fakultät Universität Zürich Winterthurerstrasse 190 ZürichCH-8057 Switzerland; c Excelsus Structural Solutions, Parkstrasse 1, VilligenCH-5234, Switzerland; University of Warsaw, Poland

**Keywords:** quantum crystallography, DFT benchmarking, crystal structures, accurate structure-specific restraints

## Abstract

There is a need for fast, efficient and accurate solid-state structure optimization for imprecise crystal structures (‘augmentation’) for subsequent property prediction in the pharmaceutical industry. Crystal structures from single-crystal X-ray, 3D electron or powder diffraction are widely available but require augmentation to the same quality level for comparative studies. Properties can be best calculated when the level of theory is alike and the accuracy, as well as the precision, are high. Moreover, the size of molecules and the complexity of structures encountered in pharmaceutical research are increasing. Efficient procedures are thus required that can also treat structures with disorder and several molecules in the asymmetric unit of the unit cell. Hence, we investigated whether ‘molecule-in-cluster’ (MIC) computations [Dittrich *et al.* (2020). *CrystEngComm***22**, 7420–7431] can reach the accuracy of full-periodic (FP) computations. Selected quantum mechanical methods are assessed. The evaluation criterion is how well the structures of 22 very low temperature high-quality structures are reproduced. Computational efficiency is also considered. A novel approach to evaluating the accuracy of quantum mechanical predictions is enforcing computed structure-specific restraints in crystallographic least-squares refinements. To complement this approach, root mean square Cartesian displacements of computed and experimental structures were also compared. Analysis shows that (*a*) MIC DFT-D computations in a quantum mechanics/molecular mechanics (QM:MM) framework provide improved restraints and coordinates over earlier MIC GFN2-xTB computations, (*b*) increasing QM basis-set size in MIC QM:MM does not systematically improve computations, and (*c*) the choice of DFT functional is less important than the choice of the basis set. Overall, MIC computations are an accurate and computationally efficient tool for solid-state structure optimization that can match FP computations to augment experimental structures.

## Abstract 2

Single-crystal X-ray structures measured at around 20 K to high resolution were refined with structure-specific restraints from quantum chemical molecule-in-cluster and full-periodic computations, which permits benchmarking levels of theory of varying sophistication. Restraints can then ‘augment’ low-quality crystal structures, with other possible applications.

## 1. Introduction

Calculating physical properties for drug design and development requires knowledge of accurate 3D solid-state structure in addition to 2D molecular connectivity, for example when estimating melting points of crystalline solids (Llinas *et al.*, 2020 ▸; Palmer *et al.*, 2015 ▸) of chemically related compounds. Melting points in turn serve in predicting intrinsic solubility (Briggner *et al.*, 2011 ▸, 2014 ▸) considering crystallinity (Abramov *et al.*, 2020 ▸), using the general solubility equation (Jain & Yalkowsky, 2001 ▸; Yalkowsky, 2014 ▸; Yalkowsky & Valvani, 1980 ▸). This is a research area where new momentum is needed (Llompart *et al.*, 2024 ▸).

**Figure 1:**
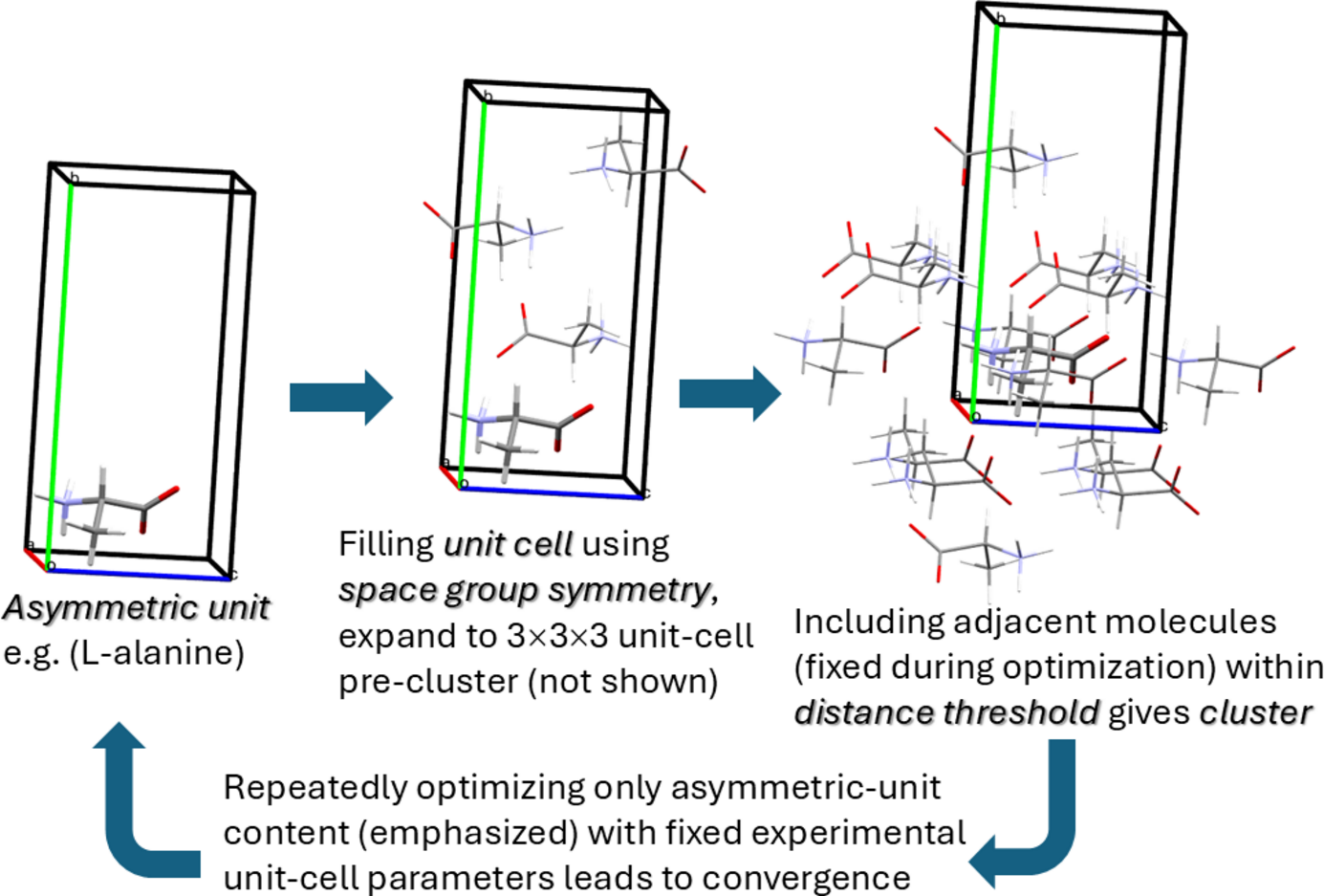
Procedure of MIC crystal structure optimization.

Structural information is widely accessible from low-cost experiments. Solid-state structures can be determined from powder diffraction (P-XRD), single-crystal electron or X-ray diffraction (ED, SC-XRD), but quality and resolution (*i.e.* precision) of results vary considerably. Structures can also be predicted *ab initio* in crystal structure prediction (CSP), albeit at extensive computational cost (Hunnisett *et al.*, 2024 ▸). A requirement for successful CSP efforts is agreement with experiment, *e.g.* in ranking polymorph energies. Reproducing SC-XRD coordinates is usually achieved by full-periodic (FP) solid-state computations (*e.g.* van de Streek & Neumann, 2010 ▸, 2014 ▸). To maximize the benefit of experimental input for computational property prediction, and for comparison in a CSP landscape, experimental crystal structures from ED and P-XRD need to be augmented to a common quality level (same level of theory, high coordinate accuracy and precision). We are interested in whether molecule-in-cluster (MIC) optimizations (Fig. 1 ▸) (Dittrich, Chan *et al.*, 2020 ▸) can provide augmentation in an economical and accurate manner.

Efficient and accurate structure augmentation would benefit applications such as using long-wavelength anomalous dispersion (Kwiatkowski *et al.*, 2000 ▸) for absolute structure determination of small-molecule structures (ongoing unpublished work), or shift calculations for NMR crystallography (Cheeseman *et al.*, 1996 ▸). We think that augmenting low-resolution structures from ED, P-XRD and macromolecular diffraction to a higher quality will lead to further useful applications. We therefore analyse and revisit 22 highly accurate low-temperature organic small-molecule crystal structures. We evaluate how well and how efficiently selected computational methods and techniques can reproduce them. Selected quality indicators of the test-set structures (see Section 2.3) are provided as supporting information (SI). In contrast to other test sets,^1^ experimental structure factors are provided. Only diffraction data where the measurement temperature was below 30 K were chosen. The experimental resolution is usually around *d* = 0.5 Å, with some exceptions (see the table in the SI). The effect of temperature can be estimated and corrected (Busing & Levy, 1964 ▸). Atomic vibrations only have a small effect on bond distances at such temperatures (see Table 1 in Section 3.1). It is therefore assumed appropriate to compare structures determined below 30 K with optimization methods explicitly not considering thermal vibration. For assessing the accuracy of selected semiempirical quantum mechanical (SQM) or quantum mechanical (QM) methods (methods in Sections 2.5.1 and 2.5.2) and density functional theory (DFT) functionals, we analyse the differences in the crystallographic *R*_1_(*F*) factor. This novel approach is enabled by tightly restraining the least-squares (LSQ) refinement using structure-specific restraints. We also calculate the root mean square Cartesian difference (RMSCD) between experiment and theory.

### 1.1. Cluster computations enable benchmarking of ‘gas-phase QM’ with SC-XRD structures

While gas-phase structures have been successfully used for benchmarking DFT, *e.g.* in Risthaus *et al.* (2014 ▸), direct comparison of atomic coordinates from DFT and SC-XRD has been mostly lacking. We emphasize the reasons why this might be. One is because non-periodic SQM or QM calculations usually rely on a Gaussian-function basis-set approximation (‘gas-phase QM’). Modelling periodic solids with such basis sets is not ideal. Therefore, for FP computations different technical approaches are in use (Jug & Bredow, 2004 ▸), *e.g.* using plane waves as the basis^2^ (Hoja *et al.*, 2019 ▸; Perdew *et al.*, 1996 ▸; Stein *et al.*, 2020 ▸; Sun *et al.*, 2015 ▸). Molecular conformations in solids often differ to the gas-phase minimum. A direct comparison of SC-XRD structures and ‘gas-phase QM’ not considering the crystal field and conformation would be comparing apples with pears. Second, SC-XRD provides the *average* structure. We need to ensure that the average structure closely corresponds to the ideal structure in the solid-state self-environment, including similarity of intra- and intermolecular interactions and excluding disorder (Dittrich, 2021 ▸). To consider the crystal field, plane wave solid-state computations necessarily assume ideal periodicity, which might not be fulfilled in a real experimental structure (Dittrich *et al.*, 2024 ▸; Spackman, 2024 ▸), and is computationally demanding. These factors can make the FP approach unsuit­able for larger molecules or complex structures of pharmaceutical interest. The latter include organic salts, cocrystals and those with multiple components in the asymmetric unit (ASU) and are frequently encountered.

Another reason SC-XRD was historically not the obvious first choice for performing comparative studies between QM and experiment is the treatment of thermal motion with anisotropic displacement parameters (ADPs) (Cruickshank, 1956*c* ▸, 1956*a* ▸, 1956*d* ▸, 1956*b* ▸). ADPs can lead to systematic differences in experimental bond distances (Busing & Levy, 1964 ▸) observed as artificial bond shortening with increasing temperature. This effect can be corrected by standard crystallographic software, *e.g. PLATON* (Spek, 2009 ▸), and is small (negligible) at exceptionally low measurement temperature. As stated, it needs to be ensured that atomic displacements are due to thermal motion and not due to other effects like disorder (Trueblood *et al.*, 1996 ▸).

The last reason SC-XRD has not been used much yet [notable exceptions being Landeros-Rivera *et al.* (2023 ▸) and Moreno Carrascosa *et al.* (2022 ▸)] for evaluating the quality of QM approaches in reproducing solid-state structure is that SC-XRD classically uses independent atom model (IAM) scattering factors. These are spherically symmetric and neglect non-sphericity of electron density ρ(**r**) due to directional bonding, lone pairs and, when applicable, *d* or higher orbitals. As a result, IAM bond distances and angles can be affected by asphericity shifts (Coppens *et al.*, 1969 ▸). Bonds are affected when features of residual ρ(**r**) are significant, biasing predominantly hydrogen positions (Dittrich *et al.*, 2005 ▸; Fabiola Sanjuan-Szklarz *et al.*, 2020 ▸; Stewart *et al.*, 1965 ▸). X-ray bond distances to hydrogen from IAM refinements are therefore found to be >10% too short compared to those from neutron diffraction (Capelli *et al.*, 2014 ▸; Dittrich *et al.*, 2005 ▸; Bacon, 1959 ▸). This is rectified when using advanced X-ray scattering factors to include fine features of ρ(**r**) (Chodkiewicz *et al.*, 2018 ▸; Dittrich *et al.*, 2013 ▸; Fugel *et al.*, 2018 ▸; Lübben *et al.*, 2019 ▸; Malaspina *et al.*, 2021 ▸), the fruit of charge density (Coppens, 1997 ▸; Koritsánszky & Coppens, 2001 ▸; Spackman & Brown, 1994 ▸; Stalke, 2011 ▸; Tsirelson & Ozerov, 1996 ▸) and quantum crystallography (QCr) (Grabowsky *et al.*, 2017 ▸) research. Experimental SC-XRD can provide the most accurate bond distances and angles for benchmarking when using non-spherical scattering factors. We use the BODD model (bond-oriented deformation density) (Lübben *et al.*, 2019 ▸) for reducing asphericity shifts. Since bond distances involving hydrogen are still less accurate and show higher standard uncertainties (s.u.’s) [for an explanation why these are not standard deviations see Schwarzenbach *et al.* (1995 ▸)] than bonds not involving hydrogen, only the latter are considered here.

Like periodic computations, QM:MM (quantum mechanics/molecular mechanics) methods and related layered cluster approaches (Dittrich, Chan *et al.*, 2020 ▸; Dittrich *et al.*, 2012 ▸, 2017 ▸; Mörschel & Schmidt, 2015 ▸; Teuteberg *et al.*, 2019 ▸) permit comparison to the experimental solid-state structure. We use the ONIOM implementation (Chung *et al.*, 2015 ▸; Svensson *et al.*, 1996 ▸, see Section 2.5.2) for this purpose. It builds on the gas-phase QM approach. Using clusters of molecules (Fig. 1 ▸), an explicit ASU environment can substitute periodic boundary conditions. Then long-range order is neglected, but symmetry is not. The influence of a long-range external field can, in addition to the explicit environment, optionally be approximated by continuum solvent models (Tomasi *et al.*, 2005 ▸). Through ONIOM, the performance of DFT functionals and basis-set choice in reproducing experimental structure can be assessed. For ONIOM, a QM ‘high-layer’ optimization (using DFT) is applied to the ASU, and an MM ‘low-layer’ treatment using a force field (FF) to the surrounding cluster molecules (Fig. 1 ▸) here. One can achieve augmentation of solid-state structures using gas-phase QM programs through clusters with these approximations. For investigating the non-disordered and still comparably simple structures of our test set, periodic computations provide reference results.

## 2. Method details

### 2.1. Benchmarking the first-principle calculation with accurate experimental X-ray structures

The dominant approach in QCr is to replace spherically symmetric IAM atomic scattering factors with non-spherical ones and thereby improve LSQ refinement. Alternatively, one can directly evaluate optimized QM coordinates via restrained SC-XRD refinement. For this purpose, cluster structures are first optimized by QM and then distances found in these optimized structures are used as structure-specific restraints in standard crystallographic refinement. Restrained LSQ refinement is expressed in equation (1), where *w_x_* are weights that usually involve a 1/σ^2^ term, σ being the s.u. of a reflection. *w*_r_ is the chosen weight for QM restraints and *D*_obs_/*T*_calc_ are the observed experimental distance/calculated target QM distance. Here we use bond distances and angles (expressed as distances, see also Section 2.5) as restraints.
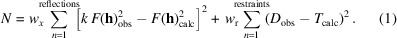
Atomic coordinates **r** = (*x*, *y*, *z*) are contained in the exponential part of the equation for calculating the structure factor *F*(**h**), *e.g.* see Dunitz (1979 ▸). Especially when artificially enforcing tight restraint targets *T*_calc_ using small restraint s.u.’s (here 0.0005 Å^2^), the crystallographic *R*_1_(*F*) factor that should be small gets *worse*. Unrestrained refinement is the reference. Differences, Δ*R*_1_(*F*) [equation (2)], can thus provide a measure of the accuracy of a chosen QM approximation method: the higher the penalty, the worse the agreement; the smaller Δ*R*_1_(*F*), the better the agreement between experiment and theory.
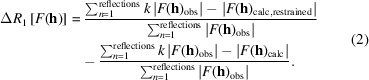
Moreover, when excluding hydrogen atoms from restraining bond distances and angles, one can pursue benchmarking efforts also using the IAM to a very good approximation. This is because coordinate and bond-distance differences for non-hydrogen atoms are small between IAM and post-IAM refinement methods (Coppens *et al.*, 1969 ▸; Dittrich *et al.*, 2007 ▸; Fabiola Sanjuan-Szklarz *et al.*, 2020 ▸) when the same set of diffraction data are evaluated. IAM bond distances for non-hydrogen atoms thus already provide high experimental accuracy. Improving on the IAM is still merited and was given appropriate attention here. Model improvements using BODD aspherical atom scattering factors (Lübben *et al.*, 2019 ▸) take deviations from the IAM into account and can conveniently be combined with restrained refinement using the well established non-linear LSQ program *SHELXL* (Sheldrick, 2008 ▸, 2015 ▸). As an alternative to Δ*R*_1_(*F*), pairwise root mean square Cartesian displacements (RMSCDs) [equation (3)] were calculated, here using the ‘Fourier’ program by van de Streek (https://github.com/JvdS147/Fourier).
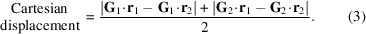
Here **r**_*i*_ are the fractional coordinates of atoms *i* in a crystal structure, and **G** is the transformation matrix from fractional to Cartesian coordinates. The RMSCD then provides an alternative measure for comparing experimentally measured and theoretically optimized sets of coordinates.

### 2.2. Requirements for experimental structures to reach high accuracy

For accurate structure determination by SC-XRD, one measures Bragg-diffraction intensities (*a*) at low temperature and (*b*) to high resolution, (*c*) minimizing sources of systematic error (Destro *et al.*, 2004 ▸; Herbst-Irmer, 2023 ▸; Larsen, 1995 ▸).

(*a*) Low temperature reduces atomic displacements, caused by atomic and lattice vibrations above the remaining zero-point motion. Atomic motion is described by the Debye–Waller factor (Debye, 1913 ▸) where the negative sign of the exponential function reduces the scattered intensity with increasing resolution through the magnitude of one isotropic or six anisotropic displacement parameters (Grosse-Kunstleve & Adams, 2002 ▸) obtained from non-linear LSQ refinement against the experimental Bragg-scattering data. Anisotropic treatment is along directions of the three reciprocal lattice vectors in units of Å^2^ and considers off-diagonal elements through a symmetric three-by-three tensor. Physical atomic displacements are thus obtained from taking the square root of the contribution in the bond direction. They are smaller the lower the temperature. We therefore chose to evaluate only data measured below 30 K. The importance of correcting displacement anisotropy in bond directions was evaluated (see Table 1).

(*b*) Reaching high resolution (sin θ/λ ≥ 1 Å^−1^ or *d* ≤ 0.5 Å) and measuring a larger number of reflections (increasingly due to core scattering) provides lower parameter s.u.’s. In non-linear LSQ refinement, s.u.’s are calculated from inverting the variance–covariance matrix. Crystal specimens with high crystal quality enabled scattering to high resolution in the test-set data (Section 2.3); intense synchrotron radiation was sometimes used to increase resolution.

(*c*) Sources of systematic errors can be extinction (Becker & Coppens, 1975 ▸), absorption (Blessing, 1995 ▸; Krause *et al.*, 2015 ▸), scan-truncation error (Lenstra *et al.*, 2001 ▸), detector characteristics [*e.g.* Zaleski *et al.* (1998 ▸)] and low-energy contamination (Domagala *et al.*, 2023 ▸) among others. Their adequate correction is mandatory for providing high-quality data (Henn, 2018 ▸). Avoidance or correction of systematic errors has been given considerable effort by the original authors in the test-set diffraction datasets chosen from the literature (Section 2.3).

When atomic displacements are small and predominantly due to thermal motion, and when SC-XRD experiments are carried out to high resolution these experiments can really provide accurate distributions of ρ(**r**) and resulting bond distances between atoms, the maxima of ρ(**r**).

### 2.3. Choice of experimental structures

**Figure 2:**
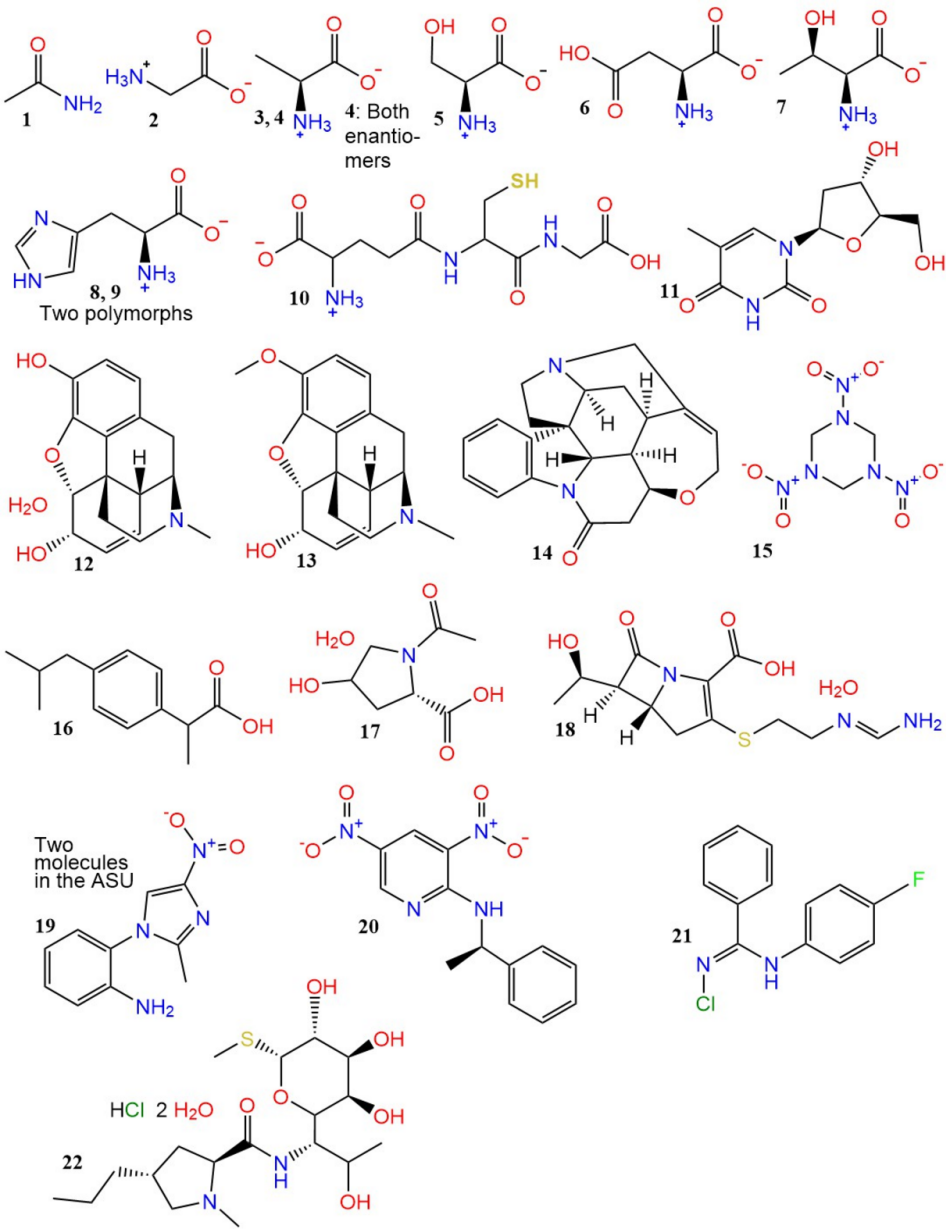
Lewis structures of 22 molecules (ASU content) of test-set structures.

For providing ground truth we chose 22 crystal structures (see Fig. 2 ▸, Lewis structures of ASU content) for which high-quality diffraction experiments were performed. Very low temperatures of around 20 K and availability of diffraction intensities were considered more important than highest resolution. Not all data reach complete coverage due to the low-temperature measurement setups, but atomic vibrations and mean square displacements in these low-temperature structures are particularly small, which minimizes libration. Molecules (ASU’s) and measurement temperatures are: (1) acetamide, 23 K (Zobel *et al.*, 1992 ▸); (2) glycine, 20 K (Destro *et al.*, 2000 ▸); (3) l-alanine, 23 K (Destro *et al.*, 1988 ▸); (4) d,l-alanine, 19 K (Destro *et al.*, 2008 ▸); (5) d,l-serine, 20 K (Dittrich *et al.*, 2005 ▸); (6) d,l-aspartic acid, 20 K (Flaig *et al.*, 1998 ▸); (7) l-threonine, 19 K (Flaig *et al.*, 1999 ▸); (8) monoclinic and (9) orthorhombic polymorphs of l-histidine, 5 K (Novelli *et al.*, 2021 ▸); (10) gluta­thione, 9 K (Hübschle *et al.*, 2018 ▸); (11) thymidine, 20 K (Hübschle *et al.*, 2008 ▸); (12) morphine monohydrate, 25 K (Scheins *et al.*, 2005 ▸); (13) codeine, 20 K (Scheins *et al.*, 2007 ▸); (14) strychnine, 25 K (Messerschmidt *et al.*, 2005 ▸); (15) RDX, 20 K (Zhurov *et al.*, 2011 ▸); (16) ibuprofen, 25 K (Kleemiss *et al.*, 2020 ▸); (17) oxaceprol [*N*-acetyl-l4-hydroxproline monohydrate], 9 K (Dittrich, Server *et al.*, 2020 ▸); (18) imipenem monohydrate, 11 K (Dittrich, Server *et al.*, 2020 ▸); see also in the SI, (19) the aniline derivative (2-methyl-4-nitro-1*H*-imidazol-1-yl)aniline with two molecules in the ASU, 10 K (Poulain *et al.*, 2014 ▸); (20) MBADNP, methyl­benzyl-amino-di­nitro­pyridine [(*R*)-3,5-di­nitro-*N*-(1-phenyl­ethyl)­pyridin-2-amine], 20 K (Cole *et al.*, 2002 ▸); (21) NCLBA [(*Z*)-*N*′-chloro-N-(4-fluoro­phenyl)­benzimidamide], 17.5 K (Destro *et al.*, 2022 ▸); and finally (22) lincomycin hydro­chloride dihydrate [dihydrate HCl salt of (2*S*,4*R*)-*N*-[(1*S*)-2-hy­droxy-1-[(3*R*,4*S*,5*R*,6*R*)-3,4,5-tri­hydroxy-6-methyl-sulfanyloxan-2-yl]propyl]-1-methyl-4-propyl­pyrrolidin-2-carboxamide], 11 K (CCDC number 2394249, https://10.5517/ccdc.csd.cc2lcdv7).

Evaluating these low-temperature diffraction data provides precise geometries with low parameter s.u.’s. None of these structures are disordered at measurement temperature; the average structure corresponds to the ideal structure. Information such SC-XRD data can provide has been discussed by, for example, Bürgi & Capelli (2003 ▸).

The set of experimental structures captures some crystallographic and chemical variety. It consists of eight zwitterionic amino acids including a pair of polymorphs, a nucleoside, the nitro-group containing explosive RDX, a non-linear optical material, the sulfur-containing oligopeptide gluta­thione with different peptide-bond binding modes, six classical drug molecules, one as an HCl salt, and the pesticide strychnine, among further compounds. Experimental diffraction intensities for some of these structures were not yet deposited. We include them as structure factors in the SI, embedded in *SHELX* type cif files which also contain restraints and BODD asphericity modelling parameters for each structure of the complete set. Coordinates for MBADNP and morphine hydrate were inverted, since their absolute configuration (Flack & Bernardinelli, 2000 ▸) was incorrect in earlier publications. For morphine hydrate, acetamide, lincomycin HCl, ibuprofen, codeine and d,l-aspartic acid, applying scale-factor corrections for post-considering integration box-size changes [or thermal-diffuse scattering (Niepötter *et al.*, 2015 ▸)] was deemed necessary and improved data quality. This became obvious when investigating their resolution-dependent scaling (Zhurov *et al.*, 2008 ▸). For acetamide the dataset retrieved is different from and does not match the resolution and quality of a now lost dataset reported in the literature (Zobel *et al.*, 1992 ▸). Nevertheless, acetamide was added to the set since it is useful for rapid testing, being even smaller than glycine. 1-(20-amino­phenyl)-2-methyl-4-nitro-1H-imidazole, gluta­thione and RDX show anharmonic thermal motion (Herbst-Irmer *et al.*, 2013 ▸) at higher temperature, but this does not much affect the very low temperature diffraction data used here for determining interatomic distances.

### 2.4. Modelling non-spherical electron density and thermal motion in experimental refinement

To evaluate restraints [equation (1)] and in parallel efficiently model ρ(**r**) in bonds and lone pairs, *SHELXL* (Sheldrick, 2008 ▸, 2015 ▸) and the BODD model (Lübben *et al.*, 2019 ▸) were relied upon. Alternative approaches, as reviewed by Korlyukov & Nelyubina (2019 ▸), were not considered but are emphasized. These are Hirshfeld atom refinement (HAR) (Capelli *et al.*, 2014 ▸; Fugel *et al.*, 2018 ▸; Jayatilaka & Dittrich, 2008 ▸) and HAR-ELMO (Malaspina *et al.*, 2019 ▸), refinements with theoretically derived multipole scattering factors (Dittrich *et al.*, 2013 ▸; Jarzembska & Dominiak, 2012 ▸) or, a new interesting development, their combination (Chodkiewicz *et al.*, 2024 ▸).

*SHELXL* non-spherical BODD refinement used anisotropic for non-hydrogen atoms and constrained isotropic displacement parameters for hydrogen atoms. The latter were multiplied by 2.4 or 3.0 rather than 1.2 or 1.5 times the *U*_eq_ value of the bonded non-hydrogen atoms (Lübben *et al.*, 2014 ▸; Madsen & Hoser, 2015 ▸). Hydrogen atoms were assigned the parameter shift of the parent atoms. This ‘riding-hydrogen’ treatment adds a small *R*-factor penalty but made refinements robust. In unrestrained refinements (UR), all non-H positions were freely refined. For restrained refinement (RR), restraints were provided to the program through an auxiliary file and evoked through the ‘+filename.rests’ option. *SHELXL* input files, and files containing the restraints, were generated with *BAERLAUCH*. A considerable number of restraints were imposed for each RR; every pair of bonded atoms was assigned a tight (restraint s.u. of 0.0005) distance restraint. Likewise, all angles were restrained as atom1–atom3 distances (angles expressed as distances). Thus, every angle not involving hydrogen atoms gave a further restraint, imposed with a softer restraint s.u. of 0.002. All other refinement options, apart from damping, were kept alike in RR and UR. Wavelength-dependence of anomalous dispersion was specifically considered for synchrotron data with values from *ShelXle* (Hübschle *et al.*, 2011 ▸). Extinction was additionally refined for glycine and lincomycin. BODD parameters excluding solvent water were assigned with the *APEX3* software (Bruker, 2019 ▸) and used throughout. BODD refinements then required three more free variables. They capture the dataset-dependent contribution of the BEDE and LONE parameters. Weighting schemes were optimized to convergence with *ShelXle* in UR and then maintained in RR. *SHELXL* cif files from UR provided reference coordinates for calculating RMSCDs for QM method evaluation. When a molecule contained more than six atoms, thermal motion analysis (TMA) was performed with the program *PLATON* (Spek, 2009 ▸). Acetamide, glycine, l- and d,l-alanine were hence excluded. TMA provided libration-corrected bonds, but not atom1–atom3 distances.

### 2.5. Computational methods

We benchmark four different theoretical methods here: (1) MIC SQM all-atom GFN2-xTB (Bannwarth *et al.*, 2019 ▸) structure optimization, (2) MIC ONIOM optimization with different methods/density functionals and basis sets for the high layer, (3) MIC ONIOM molecular orbital (MO:MO) optimization for both layers but a smaller basis set for the cluster environment, and (4) the Gaussian plus plane wave periodic-boundary approach using *CP2K* (Hutter *et al.*, 2014 ▸; Kühne *et al.*, 2020 ▸) and plane wave computations with *Quantum Espresso* (*QE*) (Giannozzi *et al.*, 2009 ▸) as a reference. MIC clusters were generated from entire ASU contents rather than from individual ASU molecules or ions. A distance threshold (Fig. 1 ▸) of 3.75 Å between the ASU-atom and the surrounding symmetry-generated ASU-molecule was chosen to generate clusters throughout. Complete ASUs were added to a cluster environment when an atom from a neighbouring molecule was within 3.75 Å.^3^ Concerning the choice of DFT-D functionals, guidance of earlier benchmarking was followed (*e.g.* Bursch *et al.*, 2022 ▸; Mardirossian & Head-Gordon, 2017 ▸; Mehta *et al.*, 2018 ▸). We share the philosophy of a focus on experiments (Mata *et al.*, 2023 ▸) for benchmarking the numerical accuracy of QM approximations.

#### 2.5.1. GFN2-xTB

Using SQM GFN2-xTB (Bannwarth *et al.*, 2019 ▸) leads to fast and computationally efficient MIC computations on CPUs. Employing the same level of theory throughout cluster-layer hierarchy can be afforded on a standard personal computer. Space-group symmetry was evaluated to set up MIC computations and their input coordinates. Only ASU atoms were optimized (Dittrich, Chan *et al.*, 2020 ▸) in a fixed surrounding of cluster molecules (Fig. 1 ▸). Input-file generation was conducted with the program *BAERLAUCH*^4^ (Dittrich *et al.*, 2012 ▸). Geometry optimization can optionally be performed evoking the ALPB continuum solvent model (Ehlert *et al.*, 2021 ▸) with water solvent and default radii at similar computational cost.

#### 2.5.2. QM:MM and MO:MO

As mentioned, QM:MM ONIOM methods separate a system into ‘high layer’ QM and ‘low layer’. The low layer level of theory is usually an MM FF, optionally with electrostatic charge embedding. We consistently applied charge embedding for the respective methods/basis sets used as reported below. Restrained fit to the electrostatic potential (RESP) charges (Bayly *et al.*, 1993 ▸) were computed for the high-layer method and assigned to the surrounding symmetry-equivalent low-layer molecules. We use the *Gaussian16* program (Frisch *et al.*, 2016 ▸) for these QM computations with the UFF force field (Rappé *et al.*, 1992 ▸) for the MM part. FF atom-type assignment in *BAERLAUCH* was automated, relying on *InvariomTool* (Hübschle *et al.*, 2007 ▸) source code. QM:MM allows comparison of selected DFT functionals and a systematic increase of the high-layer basis-set size. As an alternative to FF treatment, the low layer can also involve an MO basis-set description that is then usually less sophisticated than the high layer one. Electrostatic embedding is then not required. This is abbreviated as MO:MO and makes application of dispersion correction possible across layers – including older DFT functionals that do not already include such a correction.

#### 2.5.3. Continuum solvent models

Continuum solvent models (Tomasi *et al.*, 2005 ▸) can be used in combination with QM:MM ONIOM in *Gaussian16*. PCM (Lipparini *et al.*, 2010 ▸) and C-PCM (Barone & Cossi, 1998 ▸; Cossi *et al.*, 2003 ▸) solvent embedding for water with a default setting for optional optimization in continuous dielectric medium were evaluated. For GFN2-xTB computations with XTB, the ALPB solvent model (Ehlert *et al.*, 2021 ▸), again with water as solvent and default settings, was used. Solvent embedding can smoothen boundaries of explicit description of ASU atoms, surrounding cluster and continuum, to better approximate the crystal field. We think that invoking them is valid, since both the continuum solvent and the crystal packing share the properties of each being large assemblies of molecules that cause a response of the explicit part. In ONIOM, the role of a continuum model is not to provide a detailed description of the surrounding, but to heuristically mimic interactions of surrounding and explicit layers. A positive side effect is that permanent dipole moments of an explicit part are compensated by a continuum description. Moreover, the continuum stabilizes polarization and partial charges of the explicit part. This can be seen in more pronounced (*i.e.* larger charge separation) RESP charges for electrostatic embedding. It is not necessary to adapt permittivity ɛ for each crystal structure. ɛ for water was used throughout since the role of (partial) charge stabilization in a crystal can equally well be achieved using water as continuum solvent. This approximation induces an analogous response than a crystal field would.

#### 2.5.4. Periodic solid-state computations

Full periodic solid-state structure optimizations were performed. Model systems (Section 2.3) were either investigated with the Gaussian plus plane wave approach (VandeVondele *et al.*, 2005 ▸) using the program *CP2K* (Hutter *et al.*, 2014 ▸; Kühne *et al.*, 2020 ▸) and optimizing unit-cell parameters or with plane wave calculations using *QE* (Giannozzi *et al.*, 2009 ▸) fixing them to the experimental result. *CP2K* computations were set up with *BAERLAUCH* using one entire unit cell. *CP2K* DFT-D computations relied on the generalized gradient approximation (GGA) Perdew–Burke–Ernzerhof (PBE) exchange functional (Perdew *et al.*, 1996 ▸) with GD3BJ dispersion correction (Grimme *et al.*, 2011 ▸) and used the DZVP basis (VandeVondele & Hutter, 2007 ▸). Cutoff values for plane waves were 600 Ry with NGRIDS equal 5. For *QE*, the same PBE functional and dispersion correction were employed [for a review on dispersion correction see Grimme *et al.* (2016 ▸)]. The *QE* version was 7.4, compiled with *CUDA* acceleration and *Intel MKL*. Kinetic energy wavefunction cutoffs of 60 Ry and 240 Ry for the charge density were used for all instances with a *k*-point spacing of 0.45 Å^−1^. Norm-conserving pseudopotentials were used, namely highly optimized pseudo-dojo project (van Setten *et al.*, 2018 ▸) scalar-relativistic PBE (v0.5 stringent) pseudopotentials. *QE* computations were carried out on a Threadripper 3960X equipped with 128 GB RAM and a Quadro GV100 GPU with 32 GB VRAM. While sophisticated FP theoretical approaches are under continuous development (*e.g.* Hoja *et al.*, 2019 ▸; Stein *et al.*, 2020 ▸), GGA PBE results have proven their value in CSP and polymorph prediction/energy ranking. *CP2K* and *QE* are both freely available to academia and industry. Optimizing unit-cell parameters and atomic coordinates (as in *CP2K*) ensures reaching the global energy minimum for a given structure during *ab initio* prediction of a crystal structure. However, changed unit-cell parameters then require calculating pairwise RMSCDs with two different coordinate systems [equation (3)], so that these were not included in Fig. 9 (bottom). Since accurate experimental lattice parameters were available and are arguably preferable when this is the case, their optimization was omitted in *QE* computations. Back-conversion of *CP2K* pdb or *QE* crystallographic information file (cif) output into molecules with connectivity for further comparison and restraint generation was achieved with *PLATON* (Spek, 2009 ▸) and *ShelXle* (Hübschle *et al.*, 2011 ▸). This entailed exporting res files with *PLATON*, evoking the ‘uniq’ algorithm in *ShelXle*, and moving ASU molecule(s) into the unit cell when necessary. *BAERLAUCH* was then again used for sorting, preprocessing and converting back to cif output for RMSCD computation. To generate the same atomic sequence for comparisons with earlier MIC computations and experiment, ASU atom sorting used a combined figure of merit from extended connectivity (Rogers & Hahn, 2010 ▸), point charges, atomic masses and positional similarity.

## 3. Results and discussion

### 3.1. Thermal motion analysis

We start this section by analysing TMA corrections for bond distances of 18 molecules of the test set with more than six atoms (Fig. 2 ▸). The average difference between corrected and uncorrected bond distances is given in Table 1 ▸. As one can see, TMA correction slightly elongates bond distances by approximately 0.0005 Å or less for the low-temperature geometries used here. This value is also the restraint s.u. chosen for RRs. The value of the correction itself often barely exceeds two times its s.u., as can easily be reproduced by the *PLATON* program called with ‘calc tma’ from the SI cif files. Restrained refinement should not lead to a strong *R*-factor penalty when a target value differs within the s.u.; differences between experiment and theory are often smaller than those between varying levels of theory. For angle restraints, predicted target values need to be corrected by the cosine of the angle between two adjacent bonds, so that assigning a higher s.u. of 0.002 for them was considered appropriate. We neglected TMA corrections in the following analysis but added the feature to add/subtract average distance corrections to computed bond-distance restraints and the cosine effect on angle restraints in *BAERLAUCH*. This functionality can later provide temperature-dependent restraints for scaling predicted values, suitable for restraining, *e.g.* room-temperature experimental data with their apparently shorter bond distances.

### 3.2. Structure-specific restraints from MIC by (semiempirical) quantum chemistry

**Figure 3:**
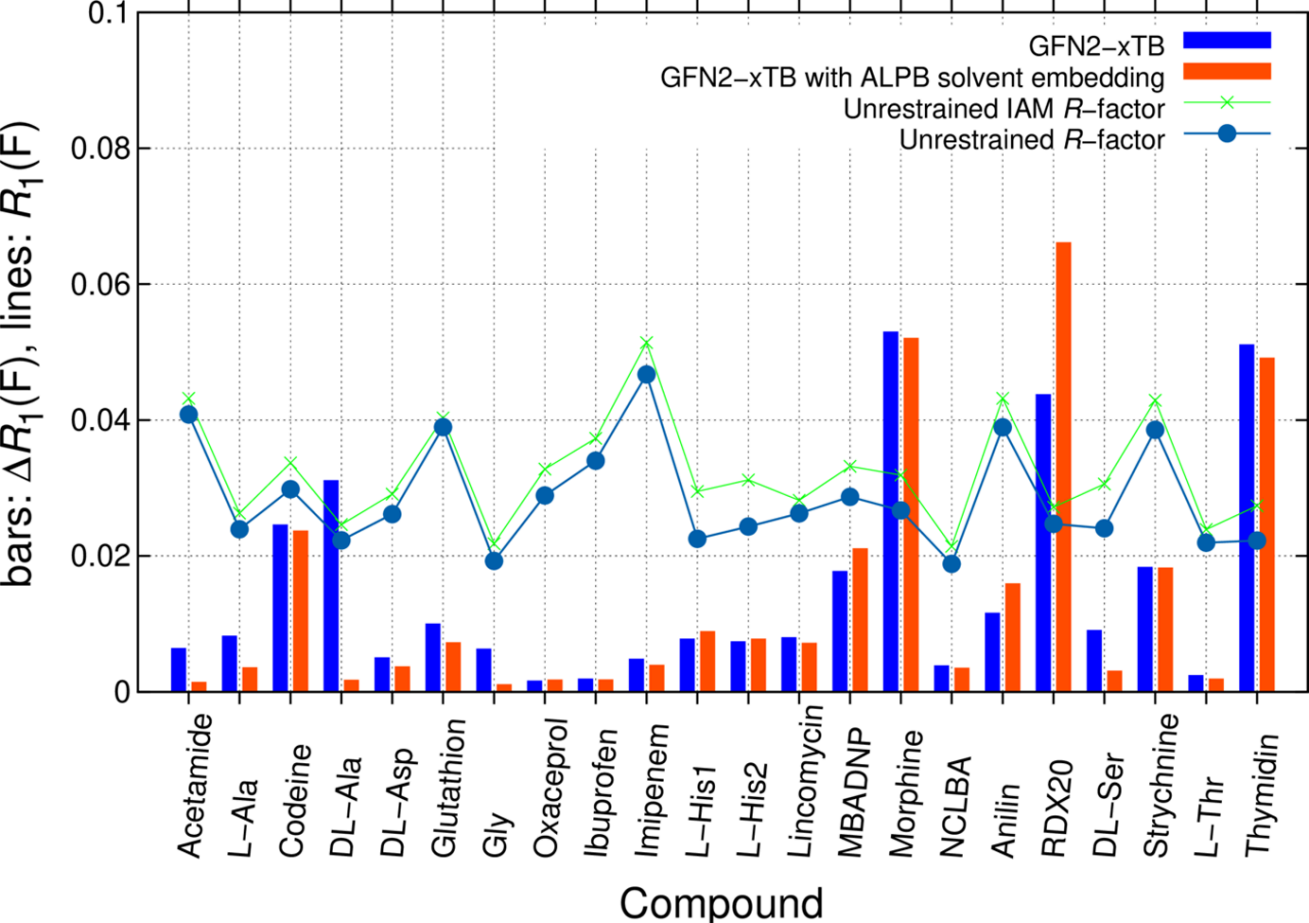
Illustration of *R*_1_(*F*) from *SHELXL* refinements with IAM (light green) and BODD aspherical scattering factors (light blue dots). A penalty Δ*R*_1_(*F*) from enforcing tight structure-specific restraints (s.u. = 0.0005 Å for bonds, s.u. = 0.002 Å for angles) from GFN2-xTB MIC optimization without (blue bars) and with (orange bars) ALPB solvent embedding is seen. The ALPB solvent model leads to better agreement for most zwitterions except l-histidine; codeine, morphine hydrate, strychnine and thymidine do not agree well at this level of theory.

**Figure 4:**
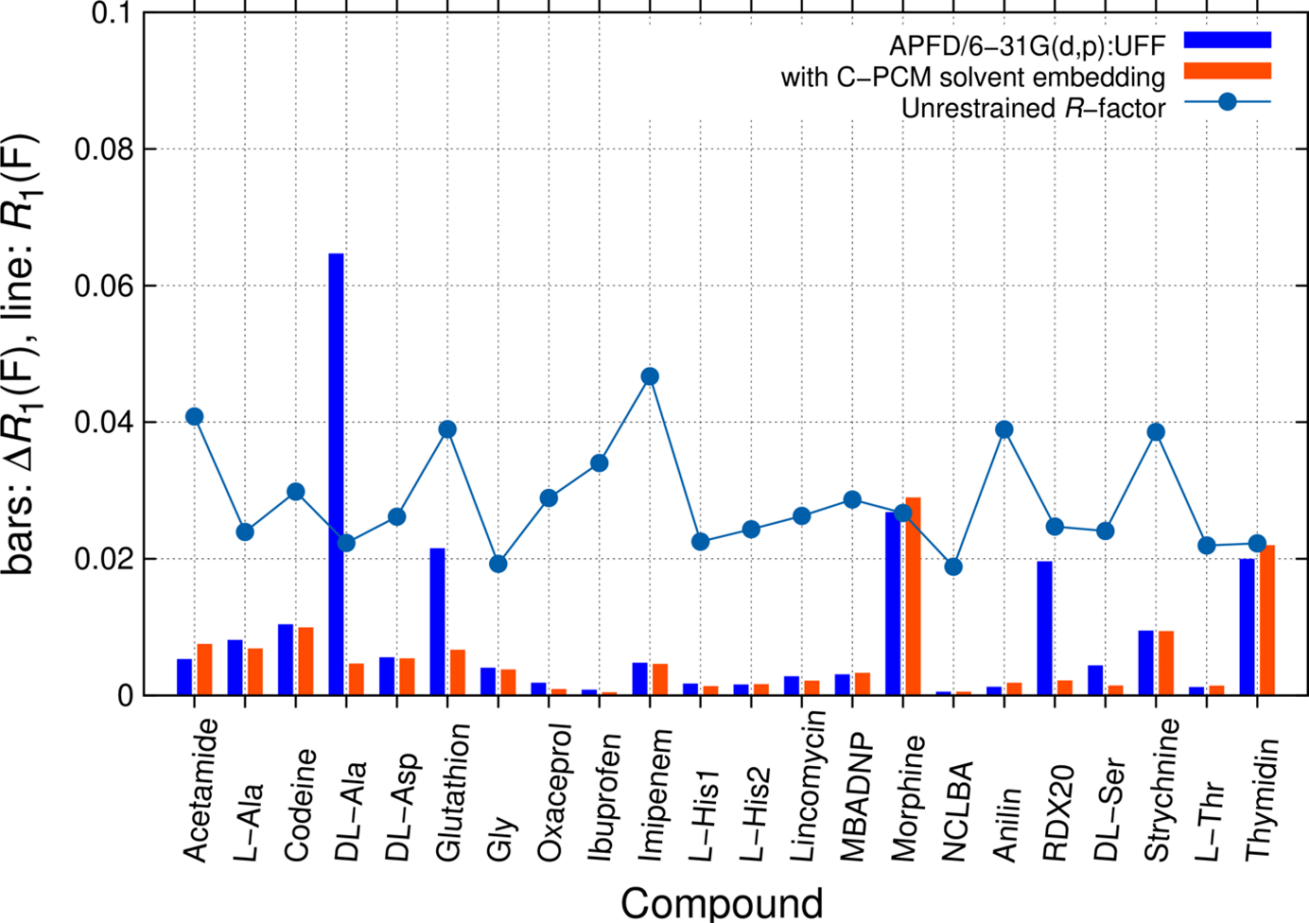
Illustration of *R*_1_(*F*) from *SHELXL* refinements with BODD aspherical scattering factors (light blue dots). A penalty Δ*R*_1_(*F*) from enforcing tight structure-specific restraints (s.u. = 0.0005 Å for bonds, s.u. = 0.002 Å for angles) from QM:MM APFD 6-31G(d,p):UFF MIC optimizations without (blue) and with (orange) C-PCM solvent embedding is shown. Using C-PCM leads to better agreement for most zwitterions; earlier compounds with high discrepancies in SQM disappear.

We continue discussing conclusions drawn from crystallographic *R*_1_(*F*)-factor differences [equation (2)] of the test-set structures, where UR are compared to RR (Fig. 3 ▸). Restraints were first generated from coordinates of two selected QM methods: SQM GFN2-xTB (Fig. 3 ▸) and QM:MM APFD/6-31G(d,p):UFF (Fig. 4). APFD was chosen as an early functional providing in-build empirical dispersion correction (Austin *et al.*, 2012 ▸) available in the *Gaussian* software.

We also evaluated GFN-FF (Spicher & Grimme, 2020 ▸) as a modern and fast FF method. Conceptually and in practise, SQM and QM restraints are superior to restraints from force fields (results not shown). This is not necessarily because of how well QM matches bond distances and angles, but because of the reliability of physics-based *ab initio* methods in improving an input solid-state structure towards the correct experimental result. This is not consistently achieved with an FF treatment. However, force fields can play an important role in stabilizing initial stages of challenging LSQ refinements in our context of restraint generation, as results can be generated almost instantaneously.

Using Δ*R*_1_(*F*) might be counterintuitive for crystallographic readers at first, since here high *R* factors do *not* mean that restraints are unsuited for practical use. Rather, enforcing restraints to be fulfilled within 0.0005/0.002 Å tightly enforces them. This leads to artificial increases in *R*_1_(*F*), here for diagnostic purposes. Relaxing restraint s.u.’s then leads to the same result as UR. UR should provide the best result, assuming the global minimum is reachable and reached. When restraints fit even within such small deviations, their quality and accuracy is confirmed. High Δ*R*_1_(*F*) highlight and emphasize coordinate or conformational differences. We can thus identify which method is best suited for reproducing the experimental structure. Evaluating diagnostic Δ*R*_1_(*F*) for all structures can then, for example, guide us how to best augment imprecise low-resolution structures in the future.

SQM GFN2-xTB computations are fastest to perform, even when repeating MIC optimizations several times to ensure convergence within the approximation of fixed experimental lattice parameters. SQM MIC optimization thus technically permits optimizing entire crystallographic databases. Moreover, GFN2-xTB optimization maintains ionization states found in experimental input. This robustness in maintaining experimental connectivity during MIC optimization has been confirmed for 732 CCDC drug-subset (Dittrich, Chan *et al.*, 2020 ▸) and numerous in-house structures. Both characteristics make the method suitable for structure validation by computational augmentation.

GFN2-xTB restraints agree well with experiment. They usually do not lead to an *R*-factor penalty in restraint refinement with a restraint s.u. of 0.01 Å or higher, can be generated even for macromolecules and help stabilize difficult refinements (Watkin, 1994 ▸). Conformers contributing to a disordered structure can be disentangled (Dittrich, 2021 ▸). While SQM optimization is usually not as accurate as the high-layer method in QM:MM (see Fig. 4 below), restraint s.u.’s can be relaxed to, for example, 0.03 Å in real-life applications and still support these use cases.

Experimental reference values of the 22 low-temperature structures are provided by UR using the BODD model, which takes asphericity into account in a convenient manner. As one can see from Fig. 3 ▸, all *R*_1_(*F*) values are low and indicate good quality of diffraction data and modelling. *R*_1_(*F*) is shown as background in Fig. 3 ▸. While BODD refinement leads to systematic improvements compared with IAM results, there is no direct correlation between Δ*R*_1_(*F*) and the UR fit. For both values, *R*_1_(*F*) and Δ*R*_1_(*F*), 10% was chosen as the upper limit in this and following similar illustrations. When comparing UR with RR, two groups of systematic deviations are seen for GFN2-xTB results. They lead to (1) systematic disagreements for zwitterions and (2) large discrepancies for more complex fused ring systems. When embedding clusters in the ALPB continuum solvent environment with water as solvent, improvements are seen for most zwitterionic structures, *e.g.* for d,l-alanine (Fig. 3 ▸).

Concerning (2), restraints from the SQM GFN2-xTB level of theory do not match well for more complex molecules with fused ring systems. This holds with and without solvent embedding. Especially high disagreements for RDX, codeine, morphine hydrate, strychnine and thymidine remain, primarily due to disagreement in angle restraints. RDX is especially sensitive to restraining and refinement even needed damping. For more accurate prediction of defined solid-state conformations of more complex molecules with their intermolecular interactions in a given experimental unit cell, the level of theory needs to be increased.

### 3.3. Structure-specific restraints from MIC by QM:MM with density functional theory

Restraint accuracy can then be further improved. For treating whole clusters of molecules on the DFT level of theory, the computational effort is still prohibitive for typical drug molecules. Using DFT methods in QM:MM approximation schemes like ONIOM renders such optimizations efficient. ONIOM 2-layer approximations, here with a QM high layer and UFF low layer, additionally allow comparison of different QM methods, *e.g.* Møller–Plesset perturbation (MP2), Hartree–Fock (HF) theory or different DFT functionals. Numerous method choices and basis-set combinations are possible.

Since the APFD functional (Austin *et al.*, 2012 ▸) provides in-built dispersion correction, we continue *R*-factor analyses with this functional, focusing on the computationally efficient 6-31G(d,p) Pople basis as available for elements up to Kr (Schuchardt *et al.*, 2007 ▸). QM:MM MIC treatment on the APFD 6-31G(d,p):UFF level of theory indeed provides better restraint accuracy (Fig. 4 ▸). Already without solvent embedding the match between theory and experiment improves for non-zwitterions RDX, codeine, morphine hydrate, strychnine and thymidine, which show significantly smaller Δ*R*_1_(*F*) values. However, zwitterionic d,l-alanine and gluta­thione now fit less well than for GFN2-xTB (with and without solvent embedding). For these two structures, QM:MM computations optimize to non-zwitterionic states in the absence of continuum solvent embedding, and pronounced disagreement is observed. One can argue that proton migration would require manual modification of the input file to avoid bias in the analysis, but we focus on the ability to accurately reproduce an experiment as quality criterion. Like for SQM, using solvent embedding, here the C-PCM model provides a remedy against proton migration in QM:MM and does not require manual intervention. It stabilizes zwitterions and better reproduces crystal conformation and ionization states. We also tried the PCM rather than the C-PCM model and the results were equivalent. PCM convergence was however not always achieved with default settings in *Gaussian* (IEF-PCM), where 5 out of the 22 molecules did not converge. Since C-PCM computations are faster to perform than PCM computations, provide similar improvements and converged directly for all 22 structures with the ‘iterative’ option, this method is favoured in the context of structure-specific restraint generation.

**Figure 5:**
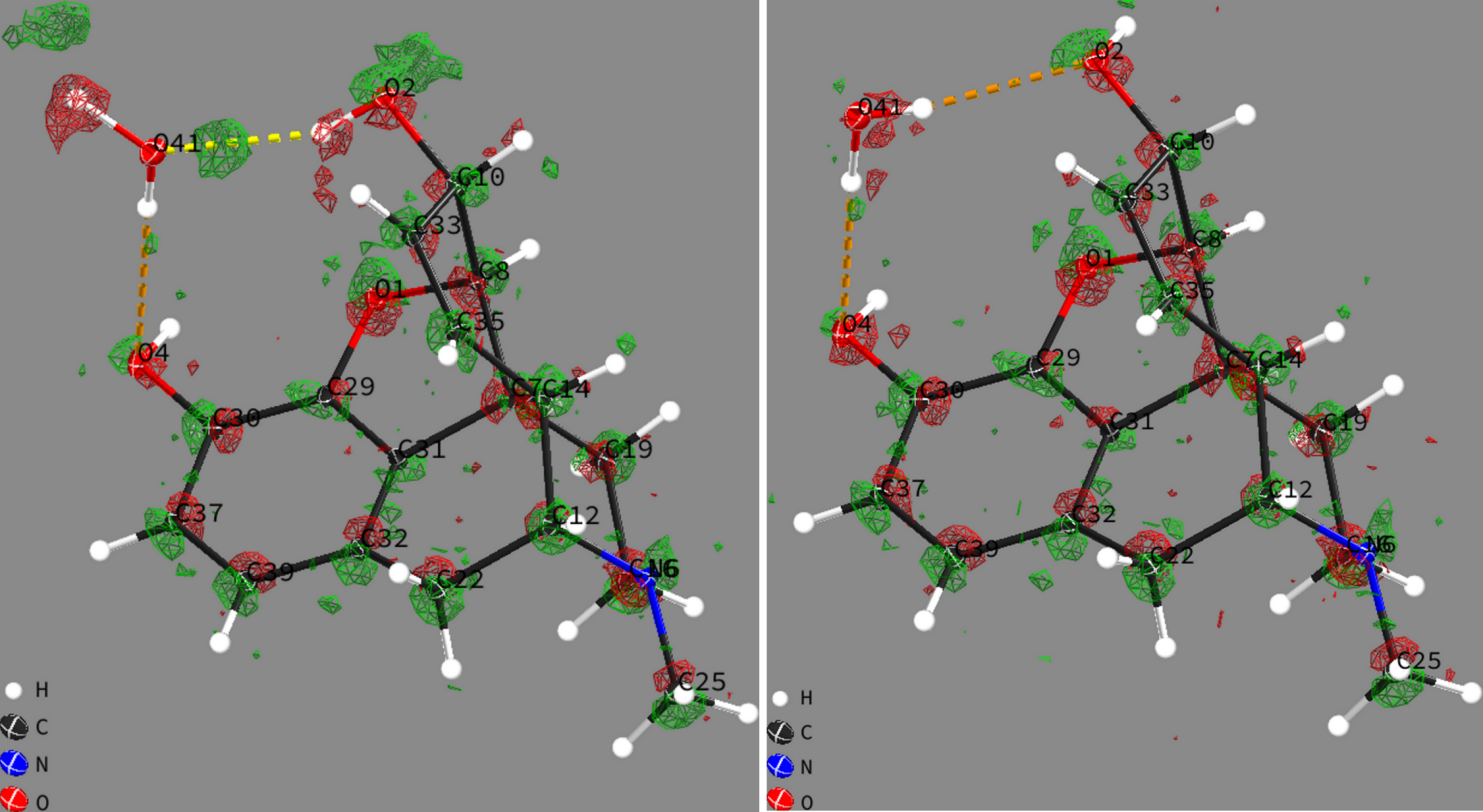
Difference electron density Δρ(**r**) in RRs of morphine hydrate plotted with *ShelXle* (Hübschle *et al.*, 2011 ▸), where water and hy­droxy hydrogens on O41 and O2 change orientation in the theoretical APFD 6-31G(d,p):UFF prediction. Green iso-surfaces (0.25 e Å^−3^) show additional ρ(**r**) whereas red iso-surfaces show less ρ(**r**) than provided by the model.

Concerning two remaining cases of comparably high Δ*R*_1_(*F*), morphine hydrate and thymidine, small structural differences can be visually identified through RR. Closer inspection leads to insight into possible dynamical behaviour of these structures. For morphine hydrate, alternative hydrogen positions in a flip-flop hydrogen bond are predicted, affecting the solvent water and a hy­droxy group (Fig. 5 ▸). This energy minimum was initialized with GFN2-xTB coordinates similar to those from experiment. The structure optimizes to a different minimum only in the C-PCM environment, with different predicted hydrogen atom positions.

For thymidine, the higher than anticipated Δ*R*_1_(*F*) reveals a predicted rotamer with different hydrogen positions in the methyl group. The predicted energetic similarity of both states should lead to rotational disorder at higher measurement temperature. It is conceivable that differences between theoretical prediction and experiment in these two structures are due to experiments providing average structure. The two highlighted structures and their equivalent energy minima affect solid-state properties via entropy. Complementing experiment by computed energies and vibrations (frequencies) is therefore attractive. Frequencies are increasingly being employed to improve ranking in CSP (Firaha *et al.*, 2023 ▸). In our opinion, archetype structures (Dittrich *et al.*, 2024 ▸) and their energy differences studied by MIC are another useful concept in this context, since they provide added flexibility compared with FP computations.

Overall, MIC QM:MM ONIOM computations reach the robustness and are not adding a prohibitive computational cost compared with SQM computations. In our opinion they are a good compromise and a step forward when higher accuracy than SQM can provide is required.

### 3.4. Effect of changing the basis set

**Figure 6:**
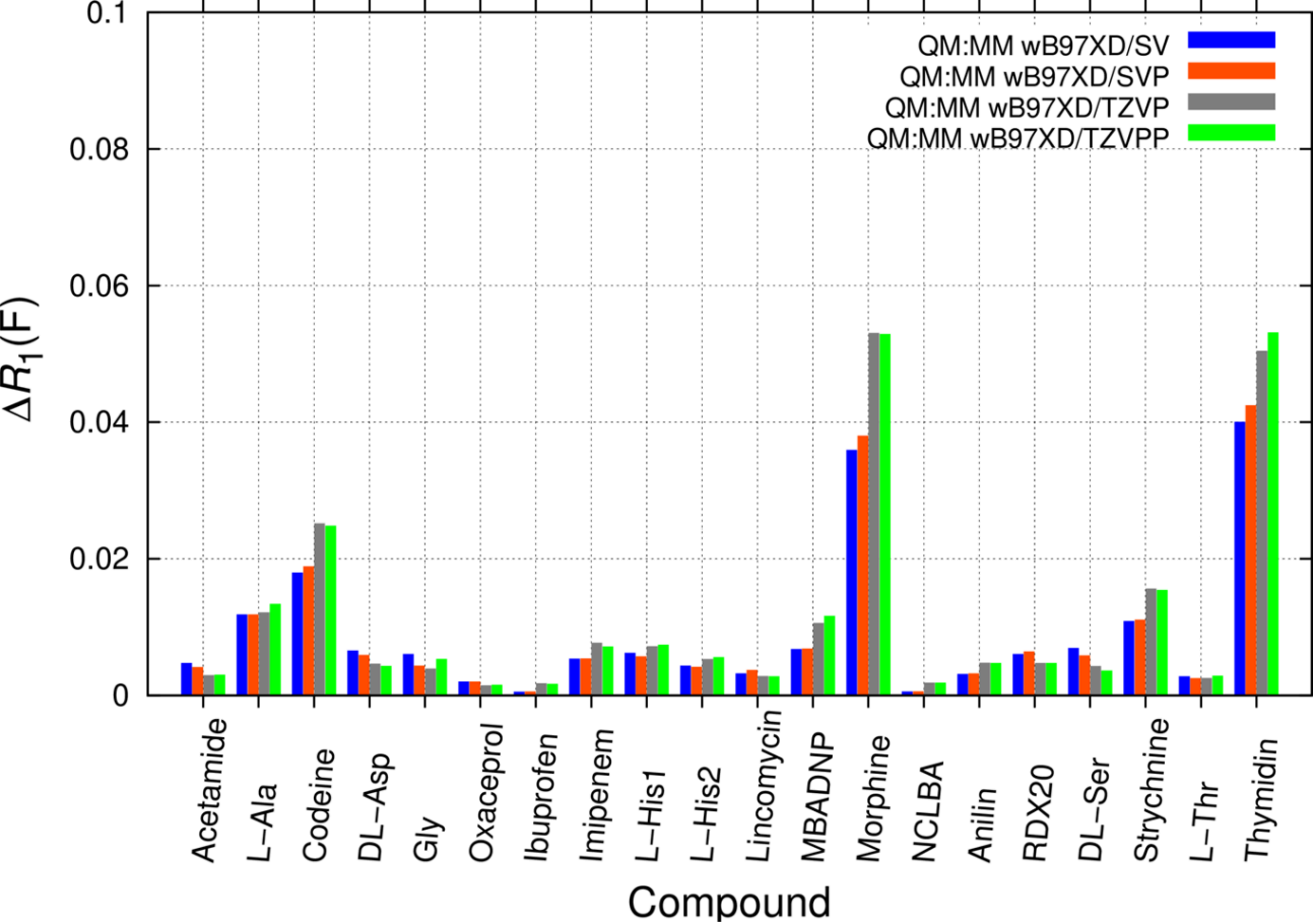
Comparison of Δ*R*_1_(*F*) from unrestrained and restrained *SHELXL* refinements with BODD aspherical scattering factors for studying increasing basis-set size. QM:MM ωB97XD/SV:UFF (blue), ωB97XD /SVP:UFF (orange), ωB97XD/TZVP:UFF (grey) and ωB97XD/TZVPP:UFF (green) ONIOM computations are investigated.

Systematically increasing basis-set size is compared next. To simultaneously try another DFT functional, ωB97XD was used, maintaining the combination with UFF low-layer treatment (Fig. 6 ▸). Several zwitterions are still included, but the cases of d,l-alanine and gluta­thione, where a change in connectivity was observed, were omitted to minimize respective bias. Continuum solvent was not used for this part of the analysis. Like the APFD DFT functional, ωB97XD incorporates empirical dispersion. The Ahlrichs-type basis set def2-SV (Weigend & Ahlrichs, 2005 ▸) was increased via def2-SVP and def2-TZVP to def2-TZVPP. For efficiency reasons, computations were initiated with APFD/6-31G(d,p):UFF coordinates for def2-SV, and subsequently with the preceding smaller basis set.

The effect of systematically increasing basis-set size on Δ*R*_1_(*F*) in highly constrained refinements only partly confirms expectations (Boese, 2015 ▸). While the def2-SV basis is, as one would expect, slightly inferior and usually leads to less satisfactory results with *R*_1_(*F*) in RR, increasing basis-set size leads to surprises. Adding polarization functions in def2-SVP leads to restraints that fit the experiment only equally good, but not better than def2-SV; def2-SVP results are similar to the APFD/6-31G(d,p):UFF combination without solvent embedding (blue bars in Fig. 4 ▸). Unexpectedly, further increasing basis-set size in the triple-ξ basis TZVP and adding further polarization functions in TZVPP does not lead to significant further improvements. It does however benefit gluta­thione^5^ (result not shown), which converges to a zwitterion also without solvent embedding with TZVPP. The apparent closeness of two gluta­thione energy minima in the same crystal packing, resulting in hydrogen migration, is interesting: it is the underlying reason for anharmonic thermal motion found in the gluta­thione structure (Hübschle *et al.*, 2018 ▸). In the bigger picture, proton transfer in states of similar energy can enable or trigger larger conformational change, which is important in many biological systems. Energies between zwitterionic or neutral gluta­thione structures remain close with def2-SVP and def2-TZVP basis sets.

It is known that bond distances become slightly shorter (Bartlett, 1994 ▸) for larger basis sets. This might be the underlying reason for the higher Δ*R*_1_(*F*) in RR. Moreover, we cannot rule out that differences in bond lengths are partly attributable to an inverse basis-set superposition error (iBSSE). While usually a calculation improves in accuracy when adding more basis functions, here we make an inverse observation. Since only the high layer partially describes parts of surrounding low-layer molecules with the extended basis sets of the central molecule(s), increasing basis-set size of the high layer might thus contribute to less good ASU geometries. As BSSE corrections [discussed in *e.g.* Mentel & Baerends (2014 ▸)] are not currently available in the ONIOM implementation used, this was not studied further. A discussion on the accuracy of Gaussian basis sets optimized for DFT (Jensen, 2017 ▸; Jensen *et al.*, 2017 ▸) could also be relevant in this context. Whereas prediction of ρ(**r**) and derived properties might benefit from more extended basis sets, using TZVP and TZVPP in this series of QM:MM computations is unnecessary for the stated aim, since there is no improvement in coordinate prediction over SVP, with positive implications on computational requirements for structure-specific restraint generation. We find that using a split valence plus polarization basis set like SVP or 6-31G(d,p) is sufficient for the stated aims.

### 3.5. Effect of changing the QM method and functional in QM:MM, MO:MO treatment

**Figure 7:**
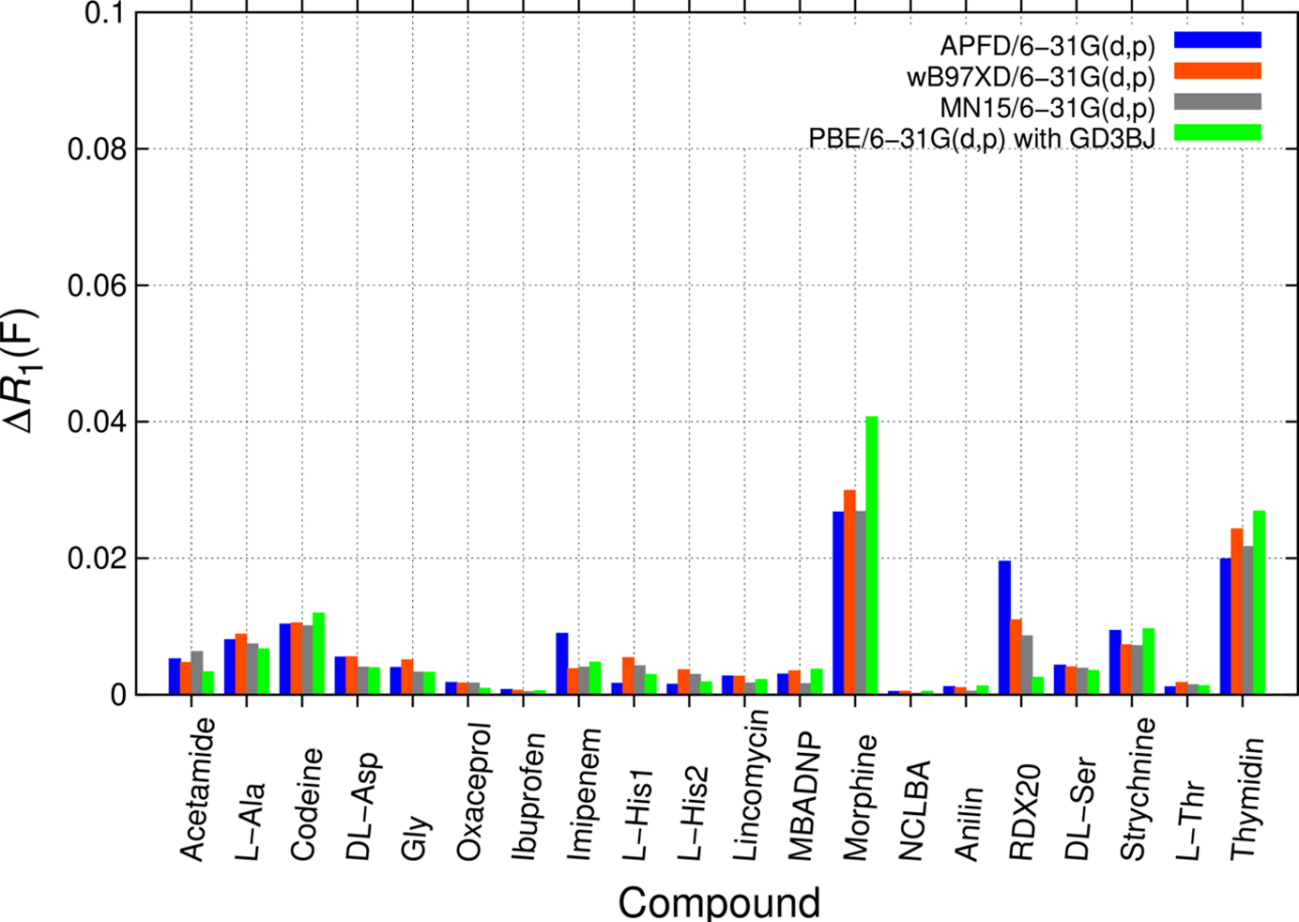
Comparison of Δ*R*_1_(*F*) from unrestrained and restrained *SHELXL* refinements with BODD aspherical scattering factors for studying influences of the DFT functional with dispersion correction in a QM:MM approach.

It was next tested if better-matching bond distances can be obtained by changing QM methods or DFT functionals (Fig. 7 ▸), starting from optimized GFN2-xTB coordinates. The following comparison again relied on the small but efficient 6-31G(d,p) basis set for minimizing potential iBSSE and the UFF force field. Reverting to HF, manually adding the GD3BJ dispersion correction [*i.e.* an ‘HF-1c’ rather than the preferrable HF-3c approach (Sure & Grimme, 2013 ▸)] did not improve matters and convergence behaviour was bad. Adding solvent embedding to HF, but not empirical dispersion, led to convergence consistently. Since solvent embedding leads to higher computational cost than adding dispersion, there is no clear gain and results are not shown. Adding dispersion correction to HF treatment has recently found use in the study of larger systems (Altun *et al.*, 2019 ▸) in a QM:MM-like framework. Convergence problems not seen in DFT-D computations were also observed for MP2 calculations without dispersion correction, where zwitterion proton transfer was, like for ‘HF-1c’, even more problematic than for DFT-D. Hence both HF and MP2 are not deemed the first choice for MIC computations for deriving structure-specific restraints in the current framework.

The next functional discussed is the pure PBE functional (Perdew *et al.*, 1996 ▸). PBE computations were found to provide similar results to the above-mentioned (Fig. 4 ▸) APFD DFT-D functional. Functionals are now all compared without solvent embedding with the same 6-31G(d,p) basis set, omitting zwitterionic/neutral d,l-alanine and gluta­thione. The PBE combination with added GD3BJ dispersion (Grimme *et al.*, 2011 ▸) can thus be used as an alternative to the more recent functionals directly incorporating it (Fig. 7 ▸). ωB97XD (Chai & Head-Gordon, 2008 ▸), as well as the most recent Minnesota functional MN15 (Yu *et al.*, 2016 ▸), perform in a similar manner to the two other functionals (Fig. 7 ▸). The influence of the functional on Δ*R*_1_(*F*) is similar to the systematic effects seen for changing basis set, but unsystematic. Hence, no clear recommendation for a ‘best functional’ emerges. Since studying the best choice of method, functional and their combination with basis sets is an extensive task [see *e.g.* Mardirossian & Head-Gordon (2017 ▸)], extended comparisons are out of scope.

**Figure 8:**
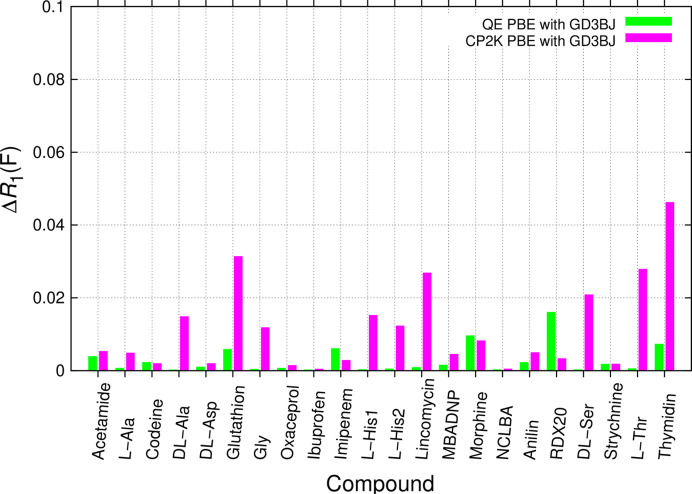
Comparison of Δ*R*_1_(*F*) from unrestrained and restrained *SHELXL* refinements with BODD aspherical scattering factors. The effect of restraints from computations using periodic boundary conditions using *CP2K* GPW (green, unit cell optimized) with the DZVP basis and D3/GD3BJ dispersion correction or *QE* plane wave PBE computations (magenta, unit cell fixed) is probed.

Next MO:MO ONIOM computations initiated with optimized APFD/6-31G(d,p):UFF coordinates are touched upon. Using MO:MO, here APFD/6-31G(d,p):APFD/STO-3G^6^ provides a systematic route for further improvements over QM:MM treatment. However, we do not see a convincing improvement from MO:MO compared with QM:MM treatment with charge embedding: (1) we encountered convergence problems and (2) a lack of computational efficiency is another factor. While MO:MO treatment could be combined with solvent embedding, this would further increase computational effort. We therefore consider MO:MO impractical for MIC optimizations at the current stage. For increasing accuracy, FP computations appear to be more attractive (see Fig. 8 ▸). Despite improvements seen in selected structures, *e.g.* for morphine hydrate, neither MO:MO treatment nor increasing basis-set size/flexibility appear to be a good use of computational resources. To improve MO:MO efficiency, it would be interesting to limit low-layer MO treatment to hydrogen bond donor/acceptor atoms and their local environment. Then a similar basis to the high could be afforded for the low layer. Automated assignment of link atoms is however currently beyond the capabilities of preprocessing tools.

### 3.6. Probing full-periodic computations for generating structure-specific restraints

**Figure 9:**
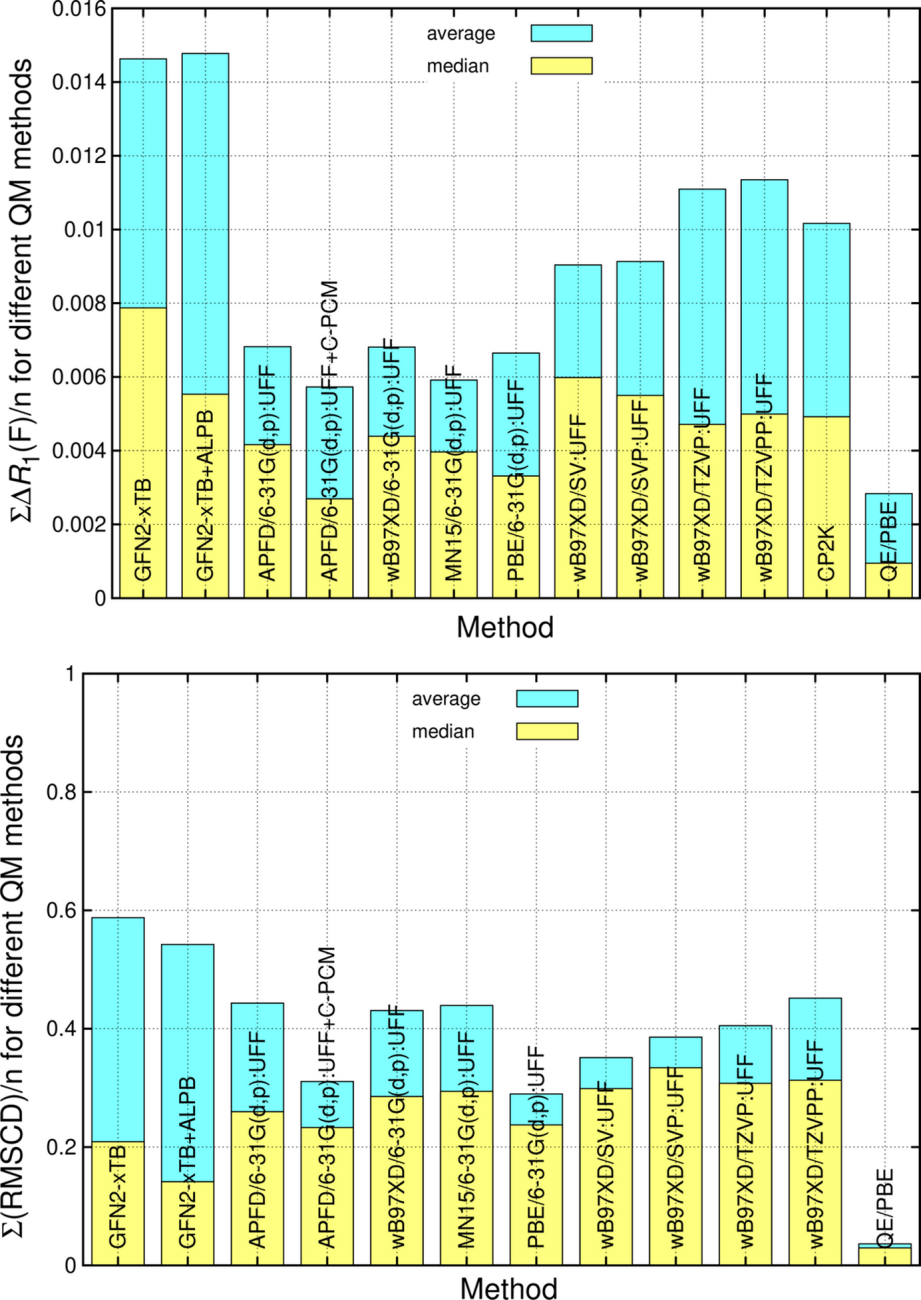
Comparison of the average and median Δ*R*_1_(*F*) (top) as well as RMSCD values (bottom) between experiment and theory for 20 structures (excluding d,l-alanine and gluta­thione) of the test set permits direct comparison of overall method performance.

Fig. 8 ▸ shows results from full periodic *CP2K* Gaussian plus plane wave as well as *QE* plane wave computations. Ionization states were maintained after reaching convergence. While there are also compounds with larger discrepancies in RR, their severity in terms of the effect on Δ*R*_1_(*F*) is less pronounced in FP than for MIC SQM or QM:MM without solvent embedding. *QE* performs especially well. *CP2K* Δ*R*_1_(*F*) results are comparable to MIC QM:MM when solvent embedding is included (Fig. 9 ▸, top). *CP2K* always performs cell optimization, *QE* optionally permits to choose. We note that FP DFT-optimized unit cells can deviate considerably from the high-quality low-temperature experimental input. This effect of using DFT-D has been discussed earlier for solid-state forms of alanine (Caetano *et al.*, 2024 ▸). We emphasize significant unit-cell changes for glycine using *CP2K* and see similar effects for exemplarily adjusting them for l-alanine with *QE*. We therefore chose not to optimize unit cells in *QE*. Distinctive program philosophies in *CP2K* and *QE* forbid concluding that differing Δ*R*_1_(*F*) results in Fig. 8 ▸ are only due to fixing the unit cell. In *CP2K* all *Z* (multiplicity) number of molecules in the unit cell are optimized independently. While the unit-cell metric of the crystal system was maintained in *CP2K*, the program philosophy is to not impose symmetry, which amounts to performing computation in the space group *P*1. Moreover, we chose to re-convert only the last set of atoms in *CP2K*, corresponding to a whole ASU. Averaging over all *Z* sets of symmetry-equivalent coordinates might have given superior results.

An inconvenience in analysing FP computations is the need for re-converting output to generate structure-specific restraints with molecular connectivity. To do so, one needs to re-establish the same atomic sequence, after shifting atoms/ions/molecules into the unit cell. Moreover, for the chosen RMSCD calculation, the same symmetry-equivalent position in the unit cell is required. For morphine hydrate and thymidine the starting coordinates might introduce bias. Despite such inconveniences and possible bias, the *QE* plane wave approach did provide the most accurate set of restraints in our study and morphine and thymidine hydrogen positions agree with the experiment. With respect to computational efficiency, we want to emphasize the more widespread use of GPUs today, which make FP, here especially *QE* computations, an excellent choice for non-disordered structures.

### 3.7. General recommendations for structure-specific restraint generation

GFN2-xTB MIC would be our choice for fast and robust optimizations, covering larger molecules, or structures with more than one molecule in the ASU. QM:MM MIC then provides increasing accuracy over GFN2-xTB. DFT functionals that include a dispersion correction are preferred for accuracy and practical convenience. For zwitterions, solvent embedding should be chosen in both SQM and QM:MM. For salts and hydrates in the test set, solvent embedding was not required. An important aspect is that cluster computations can provide restraints ‘molecule by molecule’, *i.e.* MIC by MIC. They can thus be applied for sequential optimization of complex structures. Including polarization functions did not show a clear benefit but is still recommended in the DFT basis-set description both for gas-phase QM cluster and GPW computations. When highest accuracy is required, full periodic computations can provide it. Their higher computational effort on CPUs can be circumvented when GPUs are used. MIC is expected to be considerably faster when one tests the same system on the same hardware/software.

### 3.8. Average root mean square Cartesian displacements and Δ*R*_1_(*F*)

The comparison of crystallographic *R*-factor differences Δ*R*_1_(*F*) from tightly RRs/URs provides quantitative results familiar to crystallographers. It allows studying each structure individually, thus enabling the identification of discrepancies. In addition, the average and median can be plotted (Fig. 9 ▸, top). Identification of sites of disagreement in difference electron density maps is possible (Fig. 5 ▸). Equivalent results can, to a large degree, be obtained by directly comparing coordinates of unrestrained experimental structures with optimized ones through the root mean square Cartesian displacements (RMSCDs). The RMSCD (van de Streek & Neumann, 2010 ▸) and RMSCD average/median provides (Fig. 9 ▸, bottom), like Δ*R*_1_(*F*), a single figure of merit for each structure, which is on the overall same scale. However, this requires the same connectivity and equivalence of ASU symmetry for a correct comparison; d,l-alanine and gluta­thione were therefore excluded from the following comparison, but morphine hydrate and thymidine were not. The median should be considered, because single outliers strongly influence the average. To calculate structure-specific RMSCD values we follow the approach and use the code by van de Streek & Neumann (2010 ▸), where hydrogen atoms are omitted. Since experimental unit-cell parameters are maintained in MIC and *QE* optimization, but not in *CP2K*, results for *CP2K* are systematically higher and are therefore not included. Earlier findings are confirmed by RMSCD analysis: for the average, QM:MM is superior to SQM, but for the zwitterion-heavy test set, the QM:MM median is inferior to the faster GFN2-xTB approach, especially when GFN2-xTB is combined with ALPB solvent embedding. QM:MM improves when C-PCM solvent embedding is added to QM:MM treatment. Continuum solvent models lead to a visible improvement for both SQM and QM:MM. Using a larger basis set does not reduce ∑(RMSCD)/*n* (*n* = 20). The APFD/6-31G(d,p):UFF method/basis set combination performs similar to ωB97XD, MN15 and PBE (+GD3BJ) functionals with the same basis. Single disagreements (*e.g.* RDX) strongly influence the outcome. A higher number of *n* would be needed for reliably probing functional performance. Although not directly comparable due to the local shift of ASU molecule(s) within a fixed unit cell in MIC, *QE* provides benchmark results and performs best overall. RMSCD analysis (Fig. 9 ▸, bottom) thus fully confirms recommendations from the *R*-factor analysis in Section 3.1 (Fig. 9 ▸, top).

## 4. General discussion

### 4.1. Discussion of modelling continuum solvent and crystal growth

New solid-state forms and polymorphs are often obtained by changing the dielectric constant of crystallization media (*e.g.* water, di­chloro­methane) to less polar solvents, or vice versa. Restraints for molecules with a small dipole moment show a tendency of a less good fit in RR (Figs. 3 ▸ and 4 ▸) with solvent embedding than zwitterionic molecules with their large dipole moment. This leads to the speculative aspect that molecules in crystals might maintain or ‘memorize’^7^ crystallization conditions in the solid at least to some degree, following solution pre-organization of relevant molecular conformations during crystallization. After isolation from the mother liquor, crystal packing leads to an energy barrier with respect to melting (liquid) or solid (amorphous) states. Crystals can then be maintained in time and space – if the environmental change does not exceed the barrier. Only once it does can new packings, amorphous states, liquids or gaseous states form. Predicting how to make a particular polymorph is thus not obvious, as illustrated by the recovery of crystals grown under high-pressure conditions (Fabbiani *et al.*, 2009 ▸), or those that easily incorporate or lose solvent under ambient conditions. The fact that some forms can only be made under special conditions emphasizes the need to calculate energies for non-periodic systems of local, and increasing longer-range order, in modelling. Cluster computations could become relevant to understanding crystal growth in this context.

### 4.2. General consideration and computational efficiency

MIC or FP structure optimization conceptually amounts to further crystal-structure refinement without experimental Bragg intensities, using *ab initio* QM minimization as an orthogonal procedure to LSQ. We consider this useful in many circumstances. Structure-specific restraints can add independent information to experiments every time LSQ gets stuck in a false or local minimum or provides inaccurate or ambiguous results. This applies when there is a discrete substructure hidden in the average structure, or in general when diffraction experiments are imprecise. Then they do not provide the information required for elucidating accurate coordinates. Resolving disorder as an overlay of archetype crystal structures (Dittrich, 2021 ▸) provides an example application where MIC computations are well suited (Spackman, 2024 ▸). Structure validation and augmentation including accurate hydrogen-atom placement for diffraction techniques – adding electron and powder diffraction to SC-XRD – is an application where both types of computations, MIC and full periodic, contribute.

The emphasis of this study is coordinate differences between the most accurate experimental low-temperature and QM calculated structures. Are there systematic errors common to experimental data collected by diverse research groups with different diffractometers? Does the domain structure of a crystal or does anomalous dispersion add contributions to atomic displacements, in turn systematically affecting coordinate accuracy? Can we reduce coordinate differences by improving the level of theory up to, for example, CCSD(T), or can machine learning help provide reference QM results from less costly computations, for example, as in Ramakrishnan *et al.* (2015 ▸)? Discussing, investigating and explaining root causes of discrepancies should be continued to bring theoreticians and experimentalists together for improving numerical prediction accuracy.

While we have made empirical statements about computational efficiency of MIC and FP computations, we do not provide a benchmark of overall CPU (or GPU if applicable) process time. This is because computations were performed on different hardware, including a variety of precompiled and not specially optimized programs in combined workflows. A full numerical benchmark is therefore, also considering the legal perspective, out of scope.<!?tpb=-12pt>

## 5. Conclusions and outlook

High-resolution very low temperature SC-XRD is a valuable source of information. We tried to reproduce experimental coordinates of a test set of 22 structures by computation, mostly fixing lattice constants. The main evaluation criterion was the accuracy of structure-specific restraints on bonds and angles. A penalty in Δ*R*_1_(*F*) imposing them in refinement and RMSCD coordinate differences were our diagnostic tools. Least-squares refinement invoked BODD aspherical atom scattering factors. Thermal smearing was considered a source of disagreement between experiment and theory. It was found to be unimportant for bond distances of structures measured at exceptionally low temperatures below 30 K. Selected QM methods were evaluated. Structure-specific restraints from semi-empirical, QM:MM and MO:MO MIC computations and periodic plane wave energy minimizations were compared. Especially semi-empirical GFN2-xTB computations are fast and effective. For the more accurate QM:MM DFT approach, a good compromise considering robustness, computational effort and performance in reproducing experimental structure appears to be, for example, the APFD or ωB97XD DFT functionals. Proton migration for zwitterionic structures is prevented by solvent embedding (ALPB for SQM and C-PCM for QM:MM). For QM:MM, a split-valence basis set with polarization functions and charge embedding are recommended. Extended basis sets (*e.g.* TZVPP) are counterproductive for accurately reproducing experimental crystal structures in cluster computations. MO rather than an FF treatment in the ONIOM scheme is not recommended, since it requires considerable additional computational effort.

Probing QM by restraints with a statistically more significant number of low-temperature structures could in principle also be performed with gas-phase computations when conformations in solid and gas phase are very similar.

Use cases for applying computed structure-specific restraints include augmenting imprecise low-resolution data or low-quality refinements. This applies, for example, to refinement of electron diffraction data approximated with kinematical scattering, or structures solved by simulated annealing from powder X-ray diffraction. Computational augmentation can then provide the structural quality of SC-XRD.

## Supplementary Material

CIF files with BODD parameters and additional restraints files. DOI: 10.1107/S2052252525004543/pl5046sup1.zip

Supporting table. DOI: 10.1107/S2052252525004543/pl5046sup2.pdf

CCDC references: 2462421, 2462422, 2462423, 2462424, 2462425, 2462426, 2462427, 2462428, 2462429, 2462430, 2462431, 2462432, 2462433, 2462434, 2462435, 2462436, 2462437, 2462438, 2462439, 2462440, 2462441, 2462442

## Figures and Tables

**Table 1:** Difference of the average corrected and uncorrected bond distances in the 18 test-set molecules with more than six atoms

Compound	Average distance correction from TMA (Å)
Codeine	0.00027
D,L-ASP	0.00019
Gluta­thione	0.00111
Oxaceptol	0.00038
Ibuprofen	0.00024
Imipenem monohydrate	0.0014
L-Histidine (1)	0.00068
L-Histidine (2)	0.00058
Lincomycin HCl hydrate	0.00035
MBADNP	0.00052
Morphine monohydrate	0.00026
NCLBA	0.00020
Aniline derivative	0.00034
RDX	0.00043
D,L-SER	0.00029
Strychnine	0.00039
L-THR	0.00021
Thymidine	0.00036

## Footnotes

1 Though there are already benchmark sets of crystal structures composed of molecules [*e.g.* X23 (Dolgonos *et al.*, 2019 ▸)], in these and in X23 experimental structure factors are unavailable. Moreover, accuracy as well as measurement temperature of most constituting entries do not suffice for the current analysis. Concerning benchmark studies in a purely theoretical framework, where higher-level computations serve as the benchmark for lower-level results [*e.g.* Riley *et al.* (2007 ▸) and references therein], these lack an experimental reference. A new test set was needed.

2 An exception is the periodic *CRYSTAL* code (Dovesi *et al.*, 2014 ▸; Erba *et al.*, 2023 ▸), which permits direct comparison of Gaussian basis set solid-state and gas-phase QM using the *MOLECULE* (or related *MOLSPLIT*) option.

3 When splitting ASUs into molecules (or ions) in structures with more than one molecule in the ASU (‘*Z*′ > 1 structures’), fewer molecules (basis functions) end up in the cluster with a given threshold. This leads to computational speedup. However, manual input might then be required for ensuring charge balance in salts.

4 While *BAERLAUCH* was useful in efficiently generating input files, it is not required for reproducing the results of the current study.

5 Gluta­thione, which was converging to a neutral molecule, was initiated with the zwitterionic structure from GFN2-xTB ALPB optimization.

6 MIDI (Easton *et al.*, 1996 ▸) or MINI (Sure & Grimme, 2013 ▸) basis sets would be a superior choice compared with STO-3G for the low layer at similar computational cost.

7 Linguistically the term ‘memorizing’ obviously cannot apply to a solid-state form. The point is that a system’s environment can lead to a form that is conserved in the absence of the environment through an energy barrier.
