# Supplementary material for: Benchmarking quantum chemical methods with X-ray structures via structure-specific restraints
Source: IUCrJ. 2025 Jun 17;12(Pt 4):472–87. doi: 10.1107/S2052252525004543 (PMC12224080; doi:10.1107/S2052252525004543)
Supplement: Supplementary file 1 [file m-12-00472-sup1.zip › CIF_final/Supplementary information.docx]

| Compound  (CODE) | Space  group | Resolution  (d in Å-1) | Merged y/n | Measurement  temperature | Wavelength  (Sy=synchrotron) | # reflections |
| --- | --- | --- | --- | --- | --- | --- |
| Acetamide (“ACET23K”) | *R*3 c | 0.77 | N | -250 | Mo Kα | 2326 |
| L-Alanine (ALA) | *P*2_1_ 2_1_ 2_1_ | 0.46 | Y | -253 | Mo Kα | 2536 |
| Codeine (“CODEINE”) | *P*2_1_ 2_1_ 2_1_ | 0.45 | Y | -253 | Mo Kα | 7524 |
| DL-Alanine (“DLALA”) | *P*n a 2_1_ | 0.44 | Y | -254 | Mo Kα | 2786 |
| DL-Aspartic Acid  (“DLASP”) | *C*2/c | 0.37 | Y | -253 | Ag Kα | 7139 |
| Glutathione  (“GLUTATHIONE”) | *P*2_1_ 2_1_ 2_1_ | 0.55 | N | -268 | 0.5636 (Sy) | 37975 |
| Glycine (“GLY”) | *P*2_1_/n | 0.44 | Y | -248 | Mo Kα | 3823 |
| Oxaceprol hydrate  (”HYPRO”) | *P*2_1_ 2_1_ 2_1_ | 0.49 | Y | -262 | 0.5636 (Sy) | 8305 |
| Ibuprofen (“IBU”) | *P*2_1_/c | 0.45 | N | -268 | 0.3576 (Sy) | 105440 |
| Imipenem hydrate*  (“IMI”) | *P*2_1_ 2_1_ 2_1_ | 0.68 | Y | -262 | 0.4969 (Sy) | 4621 |
| L-Histidine Mono-clinic (“LHIS1”) | *P*2_1_ | 0.70 | Y | -268 | Mo Kα | 2139 |
| L-Histidine Ortho-rhombic (“LHIS2”) | *P*2_1_ 2_1_ 2_1_ | 0.60 | Y | -268 | Mo Kα | 2929 |
| Lincomycin  HCl monohydrate  (“LINCO11”) | *P*2_1_ 2_1_ 2 | 0.50 | Y | -262 | 0.4969 (Sy) | 10578 |
| MBADNP | *P*2_1_ | 0.55 | Y | -250 | Mo Kα | 7874 |
| Morphine hydrate  (“MORPHINE”) | *P*2_1_ 2_1_ 2_1_ | 0.44 | Y | -248 | Mo Kα | 9985 |
| NCLBA (“NCLBA”) | *P*3_1_ | 0.44 | Y | -256 | Mo Kα | 10199 |
| Aniline derivative  (“POULAIN”) | *P*2_1_/c | 0.45 | Y | -263 | Mo Kα | 15218 |
| RDX (“RDX”) | *P*b c a | 0.38 | Y | -250 | Mo Kα | 8058 |
| DL-Serine (“SER”) | *P*2_1_/a | 0.42 | N | -253 | Mo Kα | 5138 |
| Strychnine  (“STRYCHNINE”) | *P*2_1_ 2_1_ 2_1_ | 0.44 | Y | -248 | Mo Kα | 10230 |
| L-Threonine (“THR”) | *P*2_1_ 2_1_ 2_1_ | 0.37 | Y | -261 | Ag Kα | 5989 |
| Thymidine  (“THYMIDINE”) | *P*2_1_ 2_1_ 2_1_ | 0.45 | Y | -253 | Mo Kα | 6296 |

*Intensities embedded for this structure in the SI CIF file have been re-integrated to provide better redundancy, coverage and resolution – using the same diffraction frames as used for the earlier CIF deposition.
