# Supplementary material for: Benchmarking quantum chemical methods with X-ray structures via structure-specific restraints
Source: IUCrJ. 2025 Jun 17;12(Pt 4):472–87. doi: 10.1107/S2052252525004543 (PMC12224080; doi:10.1107/S2052252525004543)
Supplement: Supplementary file 2 [file m-12-00472-sup2.pdf]

IUCrJ

Volume 12 (2025)

Supporting information for article:

Benchmarking quantum chemical methods with X-ray structures via structure-specific restraints

Birger Dittrich, Rok Breznikar, Gianluca Santarossa, Pamela Whitfield and Henrik Moebitz

Compound (CODE)	Space group	Resolution (d in Å-1)	Merged y/n	Measurement temperature	Wavelength (Sy=synchrotron)	# reflections
Acetamide (“ACET23K”)	*R*3 c	0.77	N	-250	Mo Kα	2326
L-Alanine (ALA)	$P2_1\ 2_1\ 2_1$	0.46	Y	-253	Mo Kα	2536
Codeine (“CODEINE”)	$P2_1\ 2_1\ 2_1$	0.45	Y	-253	Mo Kα	7524
DL-Alanine (“DLALA”)	*P*n a 2_1	0.44	Y	-254	Mo Kα	2786
DL-Aspartic Acid (“DLASP”)	*C*2/c	0.37	Y	-253	Ag Kα	7139
Glutathione (“GLUTATHIONE”)	$P2_1\ 2_1\ 2_1$	0.55	N	-268	0.5636 (Sy)	37975
Glycine (“GLY”)	$P2_1$/n	0.44	Y	-248	Mo Kα	3823
Oxaceprol hydrate (”HYPRO”)	$P2_1\ 2_1\ 2_1$	0.49	Y	-262	0.5636 (Sy)	8305
Ibuprofen (“IBU”)	$P2_1$/c	0.45	N	-268	0.3576 (Sy)	105440
Imipenem hydrate* (“IMI”)	$P2_1\ 2_1\ 2_1$	0.68	Y	-262	0.4969 (Sy)	4621
L-Histidine Mono-clinic (“LHIS1”)	$P2_1$	0.70	Y	-268	Mo Kα	2139
L-Histidine Ortho-rhombic (“LHIS2”)	$P2_1\ 2_1\ 2_1$	0.60	Y	-268	Mo Kα	2929
Lincomycin HCl monohydrate (“LINCO11”)	$P2_1\ 2_1\ 2$	0.50	Y	-262	0.4969 (Sy)	10578
MBADNP	$P2_1$	0.55	Y	-250	Mo Kα	7874
Morphine hydrate (“MORPHINE”)	$P2_1\ 2_1\ 2_1$	0.44	Y	-248	Mo Kα	9985
NCLBA (“NCLBA”)	$P3_1$	0.44	Y	-256	Mo Kα	10199
Aniline derivative (“POULAIN”)	$P2_1$/c	0.45	Y	-263	Mo Kα	15218

RDX (“RDX”)	*P*b c a	0.38	Y	-250	Mo Kα	8058
DL-Serine (“SER”)	$P2_1$/a	0.42	N	-253	Mo Kα	5138
Strychnine (“STRYCHNINE”)	$P2_1\ 2_1\ 2_1$	0.44	Y	-248	Mo Kα	10230
L-Threonine (“THR”)	$P2_1\ 2_1\ 2_1$	0.37	Y	-261	Ag Kα	5989
Thymidine (“THYMIDINE”)	$P2_1\ 2_1\ 2_1$	0.45	Y	-253	Mo Kα	6296

*Intensities embedded for this structure in the SI CIF file have been re-integrated to provide better redundancy, coverage and resolution – using the same diffraction frames as used for the earlier CIF deposition.
